# The complete chloroplast genome of *Typhonium giganteum* (Araceae)

**DOI:** 10.1080/23802359.2020.1797571

**Published:** 2020-07-25

**Authors:** Jinhyuk Kim, Junki Lee, Sunseong Choi, Sanghee Um, Hyojin Kim, Hyang Sook Chun, Gyoungju Nah

**Affiliations:** a Genome Analysis Center at National Instrumentation Center for Environmental Management, Seoul National University, Seoul, Korea; b Department of Food Science and Technology, Chung-Ang University, Ahnsung, Korea

**Keywords:** *Typhonium giganteum*, complete chloroplast genome, next generation sequencing, phylogenetic analysis

## Abstract

The complete chloroplast genome sequence of *Typhonium giganteum*, a species of the Araceae family, was characterized from the *de novo* assembly of HiSeq (Illumina Co.) paired-end sequencing data. The chloroplast genome of *T. giganteum* was 165,289 bp in length, with a large single-copy (LSC) region of 91,747 bp, a small single-copy (SSC) region of 22,550 bp, and a pair of identical inverted repeat regions (IRs) of 25,496 bp. The genome contained a total of 132 genes, including 86 protein-coding genes, 38 transfer RNA (tRNA) genes, and 8 ribosomal RNA (rRNA) genes. The phylogenetic analysis of *T. giganteum* with 12 related species revealed the closest taxonomical relationship with *Pinellia pedatisecta* in the *Araceae*.

*Typhonium giganteum,* also called giant voodoo lily, is a herbaceous perennial plant belonging to the Araceae family which consists of ∼70 species native to eastern and southern Asia, New Guinea, and Australia (Cusimano et al. 2010). The root tubers of *Typhonium giganteum* have been known for ethnopharmacological uses in Asia including China and Korea to treat stroke (Chi et al. 2010) and cancer (Khalivulla et al. 2019). Because chloroplast genome-based DNA barcode marker system has not been well established for many medicinal plants, including *T. giganteum,* we generated and the complete chloroplast genome sequence of *T. giganteum* using next generation sequencing. Our result will serve as a source of future barcode marker development, as well as assist further phylogenetic study of Araceae.species.

The fresh leaves of *Typhonium giganteum* were provided from Medicinal Herb Garden, College of Pharmacy, Seoul National University (http://snuherb.snu.ac.kr/) in Goyang, Korea (37°42′44.9″N 126°49′08.0″E) where the plant is maintained and used to construct the genomic library for Illumina Hiseq sequencing. Total genomic DNA was extracted from the leaves and deposited in National Institute of Biological Resources (42 Hwangyeong-ro, Seo-gu, Incheon, 22689, Korea) with collection number of NIBRGR0000622204. The high quality PE reads were assembled by CLC Genomics Workbench (ver. 10.0.1, CLC QIAGEN), followed by manual curation through PE reads mapping (Kim et al. 2015). Annotation of the complete chloroplast genome was performed with GeSeq and manual corrections (Tillich et al. 2017). The complete chloroplast genome sequence of *T. giganteum* was submitted to GenBank with the accession number of MN626718.

The complete chloroplast genome of *T. giganteum* was 165,289 bp in length with 35.6% of G + C content, comprising a large single copy (LSC) region of 91,747 bp, a small single copy (SSC) region of 22,550 bp, and a pair of inverted repeat (IRa and IRb) regions of 25,496 bp. The genome contained 132 genes including 86 protein-coding genes, 38 tRNA genes, and 8 rRNA genes.

In order to investigate the phylogenetic position, the complete chloroplast genome sequences of *T. giganteum* and 12 related species were aligned using MAFFT (ver. 7.271) (Katoh et al. 2002), followed by phylogenetic tree construction obtained from a Maximum Likelihood (ML) analysis with 1,000 bootstraps using MEGA 7.0 (Kumar et al. 2016). The phylogenetic tree exhibited the close relationship of *T. giganteum* with *Pinellia pedatisecta* in the family of *Aracae* (Figure 1).

**Figure 1.**
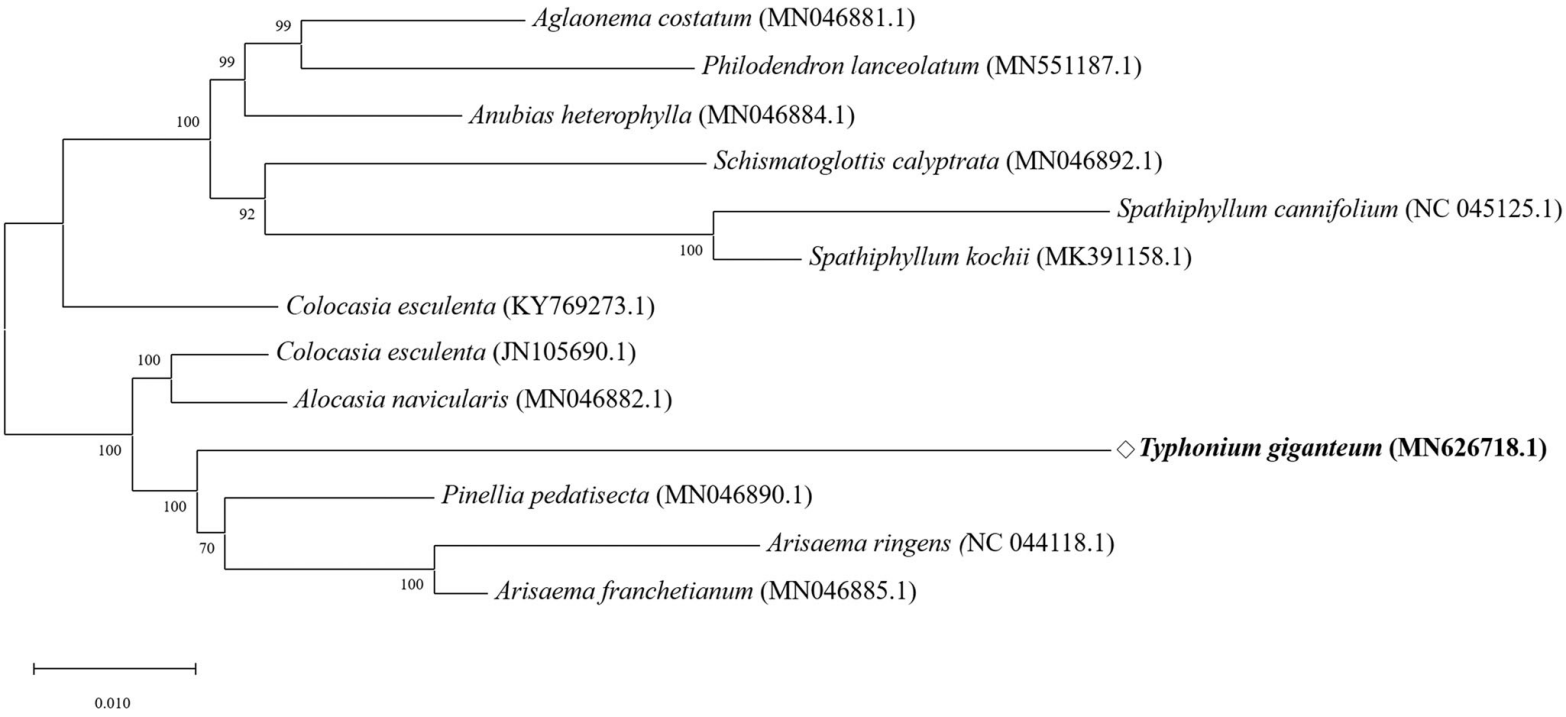
The phylogenetic tree was constructed using total chloroplast genome sequences of 12 species and *T. giganteum* using Maximum Likelihood (ML) method with bootstrap values from 1,000 replicates.

## Data Availability

The data that support the findings of this study are openly available in GenBank of NCBI at https://www.ncbi.nlm.nih.gov, reference number of MN626718.
